# Symptom experiences of patients after cardiac valve surgery: A qualitative study

**DOI:** 10.1371/journal.pone.0342597

**Published:** 2026-03-10

**Authors:** Jiajia Ban, Yuexiu Hu, Hong Huang, Ping Zhang, Shan Dong, Yujie Zhou, Ling Yuan, Yunyan Su

**Affiliations:** 1 School of Nursing, Nanjing University of Chinese Medicine, Nanjing, Jiangsu, China; 2 Department of Cardiac Surgery, Nanjing Drum Tower Hospital, Affiliated Hospital of Medical School, Nanjing University, Nanjing, Jiangsu, China; 3 Department of Oncology, Nanjing Drum Tower Hospital, Affiliated Hospital of Medical School, Nanjing University, Nanjing, Jiangsu, China; 4 Day Chemotherapy Ward，Nanjing Drum Tower Hospital, Affiliated Hospital of Medical School, Nanjing University, Nanjing, Jiangsu, China; 5 Department of Nursing, Nanjing Drum Tower Hospital, Affiliated Hospital of Medical School, Nanjing University, Nanjing, Jiangsu, China; Stamford Health System: Stamford Hospital, UNITED STATES OF AMERICA

## Abstract

**Introduction:**

Heart valve disease is a leading cause of morbidity and mortality worldwide, with surgery being the gold standard treatment.Although clinical management focuses on postoperative functional recovery, the burden of symptoms is often inadequately characterized, despite its significant impact on healthcare resource utilization and health-related quality of life.Current evidence highlights notable gaps in understanding the subjective symptom experience after cardiac valve surgery, particularly regarding the long-term evolution of symptoms and their psychosocial impact.

**Objective:**

This qualitative study aimed to systematically identify and characterize the most troubling symptom domains, specific symptoms, and symptom coping styles in patients after aortic or mitral valve replacement.

**Methods:**

We conducted semi-structured interviews with 14 adult patients (ages 26-76 years) who underwent minimally invasive or sternotomy-based valve replacement surgery at a tertiary cardiovascular center in Nanjing, China.

**Results:**

Fourteen patients (ages 26–76 years) participated in the study, from whose narratives three overarching themes and 14 subthemes were derived through thematic analysis.The analysis revealed the following:1) Physical Dimension of Symptom Experience, covering dynamic multisystem symptoms like respiratory discomfort, pharyngeal/oral issues, circulatory problems, digestive/excretory troubles, surgical pain, fatigue/weakness, and sleep disturbance; 2) Psychological Dimension of Symptom Experience, including intrinsic psychological adaptation (positive/negative) and emotional reactions to external factors (clinician interactions, family support, medical technology dependence); 3) Cognitive Dimension of Symptom Experience, involving transient ICU cognitive changes (disorientation, hallucinations) and symptom cognition (viewing pain/fatigue as inevitable, age-symptom speculation); 4) Symptom Experience and Coping Strategies in the Behavioral Dimension, encompassing passive symptom-induced behaviors and active coping (non-pharmacological, pharmacological, support-seeking).

**Conclusions:**

This study shows cardiac valve surgery patients have multidimensional, dynamic postoperative experiences. They face diverse multisystem physical symptoms, mixed positive/negative psychological states, transient ICU-related cognitive changes, and both passive and active coping strategies (with individual differences). It highlights the need for holistic, individualized postoperative care to improve recovery and quality of life.

## 1 Introduction

Cardiovascular disease (CVD) is a leading global cause of morbidity and mortality, with epidemiological trends showing persistent increases in both incidence and prevalence. Among the various subtypes of CVD, valvular heart disease (VHD) stands out as a distinct condition, characterized by structural abnormalities and neurohormonal dysregulation. These factors negatively impact biological systems and psychosocial well-being. Global surveillance data indicate that more than 42 million people worldwide are currently affected by VHD, with regional variations. In China, the prevalence ranges from 5.3% to 7.7% among adults [1]. Projections suggest a 48% rise in VHD-related disability-adjusted life years (DALYs) by 2040, driven mainly by demographic shifts towards aging populations and the growing prevalence of metabolic syndrome components [2]. These trends highlight the need for improved healthcare systems, including early detection protocols and population-level preventive strategies.

Surgical intervention remains the primary treatment for symptomatic heart valve disease, significantly improving patient prognosis [3]. However, postoperative complications—resulting from surgical trauma, extracorporeal circulation-induced inflammation, stress responses, and underlying pathophysiological changes—continue to hinder recovery and reduce quality of life. These multifactorial interactions often lead to adverse events that present a major barrier to postoperative rehabilitation [4]. Patients undergoing valve replacement frequently experience compounded physical (e.g., pain, fatigue) and psychological (e.g., anxiety, depression) symptoms that work synergistically to impair cardiovascular function, treatment response, and overall quality of life [5].

Symptoms play a crucial role in diagnosing cardiovascular diseases, assessing treatment response, and evaluating quality of life [6]. A national cross-sectional study from Denmark found that post-valve replacement patients report significantly lower quality of life compared to both healthy individuals and those with other heart conditions [7]. Therefore, improving symptom management is essential in cardiovascular disease care, as it can significantly enhance patients’ quality of life [6].

While the symptoms of VHD during its natural progression—such as angina, syncope, and dyspnea in patients with aortic stenosis—have been extensively studied [6], research on postoperative symptom evaluation remains limited and often focuses on individual symptoms. This narrow focus restricts our understanding of the full range of symptom experiences in postoperative patients and limits the development of comprehensive symptom management strategies.

The Symptom Experience Model, proposed by Armstrong in 2003, builds on the Symptom Management Model and the Theory of Unpleasant Symptoms. It provides a comprehensive framework for symptom assessment by defining the concept of symptom experience and identifying its antecedents, characteristics, and outcomes. This model enhances our understanding of symptoms by emphasizing the importance of evaluating them based on frequency, severity, distress, and meaning [8].

Qualitative research methods offer valuable insights into patients’ lived experiences, revealing psychosocial dimensions such as psychological distress and unmet social support needs. These insights can guide the development of more effective clinical management strategies. This study uses a systematic exploratory approach to investigate the postoperative symptom experiences of patients with VHD through their personal narratives. By conducting semi-structured interviews and thematic analysis, we aim to develop a comprehensive understanding of patients’ psychosocial adaptation processes and identify modifiable factors influencing their recovery. The findings will contribute to an evidence-based theoretical framework that can inform nursing interventions and rehabilitation protocols. Ultimately, this will help create patient-centered care models that address both physiological and psychosocial recovery needs.

## 2 Methods

### 2.1 Design

This qualitative study adopted purposeful sampling to recruit postoperative cardiac valve replacement/plasty patients from a tertiary care hospital in China.Semi-structured interviews were conducted to deeply explore their symptom experiences.The study followed COREQ guidelines for reporting [9].

### 2.2 Participants and setting

Study participants met the following inclusion criteria:(1)Aged ≥18 years with confirmed valvular heart disease, as per AHA/ACC guidelines;(2)Underwent elective open-heart valve surgery (replacement and/or repair) with cardiopulmonary bypass;(3)Demonstrated preserved cognitive function (MMSE ≥24) and unimpaired communication ability;(4)Provided voluntary written informed consent prior to participation.

### 2.3 Data collection procedure

Data were collected through semi-structured in-depth interviews. Interviews were scheduled for the day before the patients’ discharge from the hospital and conducted in a quiet follow-up room within the ward. All interviews were independently conducted by the researcher—a senior female nurse in the ward, concurrently pursuing a Master’s degree in Nursing. The researcher is licensed as a Registered Nurse and has been trained in the Colaizzi seven-stage analysis system.The researcher completed systematic training in qualitative research methods prior to data collection to ensure the rationality and standardization of the interview process.

Researchers provided participants with nursing care for 2–3 days prior to surgery to establish initial trust. Before the interview, they reiterated that the study was independent of clinical care to ensure that participants understood that their answers would not affect their treatment.

Before the interviews, participants were thoroughly informed about the study’s objectives and methodology. During the interviews, patients were encouraged to identify the most bothersome symptoms and describe their symptom experiences. The researcher used active listening, timely feedback, and appropriate probing questions to facilitate discussion, while also observing and documenting non-verbal cues (e.g., facial expressions, body language). Leading or suggestive language was strictly avoided throughout. Each interview lasted 20–30 minutes, and no monetary incentives were provided for participation.The sample size was determined based on the principle of data saturation, operationally defined as the point when no new major themes emerged from three consecutive interviews. To systematically evaluate saturation, thematic analyses were conducted following each interview. Data saturation was confirmed when the final three interviews (Participants #12–14) failed to generate any novel themes.

All interviews were audio-recorded using a digital recording device, transcribed verbatim, and anonymized within 24 hours.

Given the dual role of the first author (JB) as both a ward nurse involved in participants’ preoperative care and the primary researcher, this study acknowledges both methodological advantages and limitations. The clinical background facilitated rapport building with participants, enhancing the authenticity of feedback regarding nursing experiences. However, potential power imbalances inherent in the nurse–participant relationship may introduce social desirability bias, wherein participants could modify responses to align with perceived expectations.

To mitigate these concerns and ensure methodological rigor, JB maintained a reflexive journal throughout the research process. This practice adhered to phenomenological principles of bracketing, suspending prior assumptions to minimize interpretive bias. Subsequently, thematic analysis findings were presented to participants for member checking—a validation step confirming the credibility of interpretations and strengthening the trustworthiness of results.

### 2.4 The interview topic guide

A semi-structured interview protocol grounded in the Symptom Experience Model (SEM) was developed for this study. Two cardiac surgical nursing experts were consulted to create the initial draft of the interview guide. Based on two pilot interviews, the interview guide was revised as follows:

The original second question “How have these symptoms affected your life? Have they improved?” had the latter part “Have they improved?” removed, as the research team thought it might guide patients and affect response objectivity. The revised question is “How have these symptoms affected your life?”.The original fourth question “What challenges did you face during symptom management? What support helped you?” was deleted. Pilot interviews showed patients’ responses were superficial and fragmented (e.g., mentioning no special challenges or only general family care), failing to tap into core study content or contribute to the research theme.Detailed interview questions can be found in S1 File.The final core interview questions are:

(1) What physical symptoms have you experienced after surgery?
(2) How have these symptoms affected your life?
(3) What coping strategies have you used to manage them?
(4) What are your expectations for postoperative recovery?

### 2.5 Data analysis

Data were analyzed using Colaizzi’s 7-step analytical framework [10]. The researcher transcribed the audio recordings into textual data within 24 hours of each interview and cross-checked the transcripts for accuracy.Two researchers (JB, YH) conducted repeated discussions to consensus on themes and sub-themes. Details are as follows:(a) Familiarization:JB and YH listened to audio recordings and reread transcripts at least three times to gain a deep understanding of the participants’ lived experiences while refraining from early interpretation；(b) Identifying significant statements:Both researchers independently extracted statements that reflected participants’ core experiences and perceptions. Any discrepancies were resolved through discussion；(c) Formulating meanings:JB and YH coded the significant statements to formulate meanings. A third researcher (HH) was involved in discussions to cross-validate the formulations and achieve consensus；(d) Developing themes:The validated formulated meanings were grouped into meaning units. Common concepts across these units were then synthesized into preliminary themes；(e) Refining themes:The preliminary themes were refined into major themes and sub-themes. Detailed descriptions were developed and linked back to participants’ original statements. Consensus was confirmed by JB and YH；(f) Expert review:An external qualitative research expert (LY) reviewed the meaning units and themes for alignment with the data, logical consistency, and comprehensiveness. Revisions were made based on the feedback provided；(g) Member checking and final description:The final themes were returned to participants for verification (member checking). After incorporating their feedback, the themes were integrated into an exhaustive description that summarized the core essence of the lived experience.

Two authors independently conducted the initial open coding of interview transcripts using NVivo (v.15) software to facilitate systematic data management. To ensure inter-rater reliability, a randomly selected subset (28%) of the total coded units was double-coded, and inter-coder agreement was assessed using Cohen’s Kappa coefficient, calculated via the R package “irr”. The obtained Kappa value of κ = 0.89 indicated “substantial agreement” based on established benchmarks [11]. All initial coding disagreements were resolved through a structured procedure: first, the two coders discussed discrepancies within 48 hours with reference to the predefined codebook and original text, resolving approximately 92% of cases; for the remaining 8%, a senior researcher with expertise in qualitative methods was consulted to make the final decision. Relevant codes were subsequently consolidated into thematic categories, followed by an interpretive review of category content to identify patterns and relationships.Relevant codes were consolidated into thematic categories, followed by an interpretive review of category content to identify patterns and relationships.The researchers systematically established an audit trail from raw interview transcripts to coded data and final theme development, ensuring methodological rigor. The research team engaged in iterative discussions regarding initial coding frameworks, emerging category structures, and final themes to enhance credibility and verifiability. Reflexivity was strengthened through group reflections on individual experiences and field notes, ensuring critical awareness of researcher bias throughout the analysis process.Thematic evolution, using respiratory discomfort as an example, is detailed in S2 Fig.

### 2.6 Ethical considerations

The research protocol was approved by the Ethics Review Committee of Nanjing Gulou Hospital (approval No. 2025-0040-02),and written informed consent was obtained from all participants.During patient enrollment, cognitive function was preliminarily assessed using the Mini-Mental State Examination. All participants demonstrated normal cognitive function, enabling them to fully comprehend all research interview questions and independently provide informed consent.

## 3 Results

The study was conducted from March 25, 2025 to May 1, 2025.

A total of 14 patients participated in this study, with a 100% interview completion rate. The demographic and clinical characteristics of the participants are summarized in Table 1. Thematic analysis identified four main themes and thirteen subthemes:(1) Physical Dimension of Symptom Experience;(2) Psychological Dimension of Symptom Experience;(3) Cognitive Dimension of Symptom Experience; (4) Symptom Experience and Coping Strategies in the Behavioral Dimension.

**Table 1:** Characteristics of participants.

Interviewed Participant (P)	Gender	Age	Educational background	Marriage status	Residence	Occupation	surgery type	key symptoms
P01	Male	52	College or above	Male	Urban Area	Worker	MVP + TVP	Cough, Fatigue and weakness, pain, Anorexia,Cognitive Function Changes
P02	Female	46	Junior high school	Female	Urban Area	Worker	MVR + TVP	pain, palpitations, Pharyngeal Discomfor
P03	Male	44	Senior high school	18 ~ 39	Urban Area	Freelancer	MVP	Fatigue and weakness, pain, palpitations, Thirst, Family-Related Situational Responses, Clinician-Related Situational Responses
P04	Male	51	College or above	40 ~ 60	Urban Area	Business Manager	MVP	Cough, pain, Anorexia, palpitations, Oral Ulcers, Positive Intrinsic Psychological Adaptation, Clinician-Related Situational Responses, Passive Behavioral Manifestations Triggered by Symptoms
P05	Female	71	Primary school	>60	Rural Area	Farmer	AVR + MVR + TVP	Cough, Chest tightness, Fatigue and weakness, Sleep Disturbance, Constipation, abnormal urination, Negative Intrinsic Psychological Adaptation
P06	Male	76	Primary school	Primary school or below	Urban Area	Retired	MVP + TVP	Cough, Sleep Disturbance, Constipation, Positive Intrinsic Psychological Adaptation, Family-Related Situational Responses, Clinician-Related Situational Responses
P07	Female	54	Junior high school	Junior high school	Urban Area	Farmer	AVR + MVP + TVP	Shortness of breath, Sleep Disturbance, Thirst, Feeling of Pharyngeal Obstruction During Swallowing
P08	Male	26	College or above	Senior high school	Rural Area	Student	AVR + MVR	Fatigue and weakness, pain, palpitations, Negative Intrinsic Psychological Adaptation, Responses Related to Reliance on Medical Technology, Passive Behavioral Manifestations Triggered by Symptoms
P09	Male	36	College or above	College or above	Urban Area	Professional and Technical Personnel	MVP + TVP	Fatigue and weakness, pain, Anorexia, Positive Intrinsic Psychological Adaptation
P10	Male	73	Senior high school	Married	Urban Area	Retired	AVR + MVR + TVP	Cough, Fatigue and weakness, Sleep Disturbance, Anorexia, palpitations, Negative Intrinsic Psychological Adaptation
P11	Male	67	Junior high school	Single	Urban Area	Worker	MVP + AVP + TVP	Chest tightness, Fatigue and weakness
P12	Female	71	Primary school	Urban Area	Urban Area	Farmer	MVR + TVP	Cough, pain
P13	Female	52	Primary school	Rural Area	Urban Area	Farmer	AVR + TVP	Cough, Sleep Disturbance,
P14	Female	62	Primary school	Worker	Urban Area	Retired	AVR + MVR + TVP	Fatigue and weakness, Sleep Disturbance, pain, Anorexia, palpitations, Thirst, Cognitive Function Changes

Notes: AVR:Aortic Valve Replacement AVP:Aortic Valvuloplasty MVR:Mitral Valve Replacement MVP:Mitral Valvuloplasty TVP:Tricuspid Valvuloplasty

### 3.1 Theme 1: Physical dimension of symptom experience

Patients reported diverse physical symptoms following cardiac valve surgery, including respiratory discomfort, circulatory disturbances, gastrointestinal and excretory issues, fatigue, pain, and sleep disturbances. These symptoms were dynamic, fluctuating in frequency, intensity, and duration across distinct phases of postoperative recovery.

#### Subtheme 1:Respiratory discomfort.

Respiratory discomfort was predominantly reported in the early postoperative period, manifesting as cough, chest tightness, dyspnea, and throat irritation—reflecting both surgical trauma and adaptive physiological changes.

(1) Cough:

Eight patients described postoperative cough, primarily characterized as irritative dry cough. Precipitating factors included positional changes, prolonged speech, and nebulization therapy. Concurrent back pain was reported by some, with symptoms persisting 3–5 days on average and gradually resolving with recovery. Among smokers, withdrawal-related cough was a potential contributing factor.

*“Two days after being transferred out of the care unit, I felt the urge to cough. It was just a dry cough with no sputum”* (P08).

*“If I sit in the wrong position, stay in one position for too long, or talk for a long time, I have to cough immediately”* (P01).

*“Sputum couldn’t be coughed up—there was sputum, but it was difficult to expectorate, and the sputum that came out was like mucus”“After I cough, both sides of my back hurt.”* (P10).

*“The cough has lasted for four to five days, and it is slightly better now”* (P05). *“The cough symptoms are getting increasingly mild” (P06).*

*“I used to smoke. If I go days without one, I might also get withdrawal symptoms like this cough.”*(04)

(2) Chest tightness and shortness of breath

Chest tightness usually has to do with things like pleural effusion, and it comes with a general feeling of discomfort. Shortness of breath usually starts around a week after surgery—it’s a new post-op symptom for some patients.

*“ A few days ago, I felt tight in my chest. And when I did, I felt unwell all over. The doctor did an ultrasound, and there’s a little bit of pleural effusion. The doctor said this kind of thing can also cause chest tightness, and I’m much better now.” (05)*

*“Around a week or so after surgery, I started feeling a little short of breath.” (P07)*

#### Subtheme 2: Discomfort in the pharynx and oral cavity.

Postoperative pharyngeal and oral discomfort primarily involved the pharynx, oral mucosa, and swallowing-related structures, categorized into four subtypes: pharyngeal irritation, thirst, oral ulcers, and dysphagia with a choking sensation.

(1) Pharyngeal Discomfort

Manifestations included dry throat, soreness, hoarseness, and a foreign body sensation, primarily attributed to endotracheal intubation. Symptoms were self-limiting and resolved within days.

*“After the endotracheal tube was removed,I lost my voice. Subsequently, I developed hoarseness that persisted for one to two days before gradually resolving. My voice has now nearly returned to normal.”* (P02)

*“While in the intensive care unit (ICU), I experienced significant throat discomfort and a persistent sensation of a foreign object. These symptoms improved after I was transferred from the ICU.”* (P07)

(2) Thirst

Three patients reported severe thirst, characterized by an intense subjective desire for fluids, exacerbated by medically mandated fluid restrictions. Many described it as one of the most distressing symptoms.

*“The most unbearable symptom after surgery was thirst—an extreme, overwhelming dryness. I felt I could instantly finish three more bottles of water, but I had to comply with the medical team’s guidance on fluid management.”* (P03)

*“Thirst was the most severe issue. I had a constant, intense desire to drink throughout the day, yet I understood that adhering to the fluid restriction protocol was crucial for my recovery.”* (P07)

(3) Oral Ulcers

One patient developed oral ulcers causing severe swallowing pain and subsequent dysphagia.

*“I developed an ulcer in my mouth that caused extreme pain, making it impossible for me to swallow any food.” “The symptoms persisted for over ten days before significant improvement was observed.”* (P04)

(4) Feeling of Pharyngeal Obstruction During Swallowing

One patient reported a feeling of pharyngeal obstruction during swallowing postoperatively. Its main manifestation was “choking sensation while eating,” without accompanying coughing. The sensation of obstruction is directly related to eating speed and the amount of food taken in a single bite, and the symptom can be effectively alleviated by adjusting eating methods.

*“These days, I have been a bit prone to choking while eating. Suddenly, when I ate a little bit of apple, I choked.” “Sometimes I also choke when I eat,” “I don’t cough when eating, just choke.” “When I choke, I can drink some water, and then I can swallow the food down.” “I need to eat slowly, taking small bites at a time, and then I won’t choke.”* (P07)

#### Subtheme 3: Circulatory discomfort.

Four patients reported circulatory discomfort.Postoperative circulatory discomfort was primarily manifested as palpitations, encompassing tachycardia and arrhythmias. These symptoms frequently emerged in the early postoperative phase, with some cases exhibiting a correlation with a pre-existing history of atrial fibrillation.

*“After I got moved to the ward, the first two days—especially the first day—my heart was beating so fast. It was like I’d just run 1500 meters, felt like my heart was gonna jump right out of my chest. The second day was a little better than the first.”* (P04)

*“I have atrial fibrillation. When I get up and walk around, I feel a little flustered.”* (P02)

*“It feels like my heartbeat’s a bit all over the place.”* (P10)

#### Subtheme 4: Digestive and excretory issues.

The postoperative digestive and excretory problems in patients with valvular heart disease are mainly manifested as anorexia, constipation, and abnormal urination.

(1) Anorexia

Eight patients reported early postoperative anorexia, marked by reduced appetite and food intake. Some developed taste preferences or swallowing-related restrictions.

*“During the first three days after I was transferred out of the ICU, I basically had no desire to eat at all. I could only eat a little fruit and some eight-treasure porridge—those were the only things I could have a bit of; I couldn’t stand anything else.”* (P09)

*“Now my food intake is half of what it was before the surgery. Before, the total amount of vegetables and rice I ate across three meals might have been 1.5 kilograms, but now it’s probably at most 0.5 kilograms, and I might not even reach that amount.”* (P10)

(2) Constipation

Constipation is a common short-term excretory problem after surgery and can be alleviated with recovery.

*“A few days ago, having a bowel movement or urinating was difficult, but now it’s easy.”* (P05)

*“I feel that during the recovery period after surgery, having a bowel movement is quite difficult.”* (P06)

(3) Abnormal urination

In the immediate postoperative period, some patients exhibited transient reduced urine output. This condition was occasionally accompanied by subjective reports of chest tightness. A trend of spontaneous improvement in urinary output was observed alongside overall clinical recovery.

*“When my urine output is low, I feel chest tightness and then discomfort all over my body.”*

*“My urine output was also very low yesterday, but it’s okay today.”* (P05)

#### Subtheme 5: Surgery-related pain.

Seven patients reported postoperative wound pain. The nature of the pain was diverse, including cramping pain, stabbing pain, burning pain, and pulling pain. Activity, coughing, and position changes were the main triggering factors. Some patients experienced pain with temporal regularity, and there were significant individual differences in pain intensity.

*“The inside of the wound feels like it’s cramping.”* (P03)

*“When I walk, my wound hurts a little” and “When the wound is pulled, it hurts.”* (P08)

*“My wound hurts. The first time, the pain was extremely severe—it felt like being stabbed with a knife, and there was also a burning sensation.”* (P09)

*“My wound hurts every night around 12 o’clock, as well as in the morning —it’s really uncomfortable.”*(P14)

#### Subtheme 6: Fatigue and weakness.

A prevalent symptom, characterized by generalized weakness, reduced activity tolerance, drowsiness, and diminished motivation. Ten patients in this study reported experiencing fatigue and weakness.Exacerbated by blood draws, chest tightness, or psychological distress, it improved with mild activity.

*“Right now my body’s still weak and needs to recover—I can’t walk fast, and I can’t run around like I used to”* (P01);

*“After the surgery, I always feel lifeless—I don’t have the energy for anything”* (P03),

*“I often feel tired. After the surgery, when I walk—even just 70 meters—I start to feel a bit tired. And I also often feel like sleeping, so I’ll rest then”* (P08);

*“I feel fine if I don’t have blood drawn, but as soon as I do, I feel even more tired” and “When I have chest tightness, the feeling of fatigue gets worse”* (P11).

#### Subtheme 7: Sleep disturbance.

Six patients reported poor sleep: shortened duration, frequent awakenings, and light sleep, particularly in the ICU. Contributing factors included environmental disruptions and pain; pre-existing sleep issues worsened postoperatively.

*“I just lie there with my eyes closed. For almost three nights in a row, I only slept two or three hours and then couldn’t fall back asleep”* (P05)

*“I was in the ICU for two nights. After the tube was removed, I couldn’t sleep at all—every time I closed my eyes, the nurses would come to resuscitate another patient”* (P07)

*“I usually try to sleep around 8 o’clock, but I wake up again around 11 or 12. Once I’m awake, I can’t go back to sleep, and then I just end up feeling groggy. Anyway, that’s how it is—my sleep is poor”* (P10)

*“My sleep is bad. I’m not used to the bed, and I have some overall body aches that make me uncomfortable”* (P13)

### 3.2 Theme 2: Psychological dimension of symptom experience

The psychological dimension of patients’ symptom experiences encompasses two core aspects: first, the intrinsic psychological adaptation state, which arises spontaneously without direct external triggers; and second, the emotional responses directly elicited by external factors such as clinician-patient interaction, family support, and medical interventions.

#### Subtheme 1: Intrinsic psychological adaptation state.

Intrinsic psychological adaptation can be categorized into two distinct types: positive adaptation and negative adaptation, reflecting patients’ psychological resilience and distress, respectively, following surgery.

(1) Positive Intrinsic Psychological Adaptation

Positive adaptation is manifested through spontaneously generated positive emotions—such as freedom from anxiety and optimism—as well as constructive cognitions regarding postoperative recovery and self-perception. It reflects an inherent psychological resilience and a tendency toward positive thinking, which can develop independently of external support or guidance.This also reflects the interaction between physical symptoms and psychological symptoms.

*“I’ve never felt anxious or worried at all. Even when I felt unwell, I didn’t worry much.” “I’m not anxious at all—anyway, you can’t avoid it; you have to have this surgery eventually.”* (P04)

*“I just think things will definitely get better.” “It’s okay if it’s a bit slow—overall, I’m getting better day by day, and we can see hope.” “I’m very optimistic, and I cherish my life a lot.”* (P06)

(2) Negative Intrinsic Psychological Adaptation

Negative adaptation is characterized by spontaneously arising negative emotions, such as fear and psychological stress. It often stems from limited understanding of one’s symptoms, concerns about surgical risks, or internal distress caused by postoperative physiological changes, reflecting a state of psychological difficulty in adjustment.

*“Sometimes I feel a bit of chest tightness, and we don’t understand the medical field at all—so we felt a bit scared.”* (P05)

*“When my heartbeat is irregular, I feel a bit of psychological burden.”* (P10)

*“We all know that there are indeed risks after valve replacement, and my mindset was really bad during that time.”* (P03)

*“I had ventricular tachycardia (VT) twice—back then, I was really stressed out and didn’t dare to sleep at night.”*(P08)

#### Subtheme 2: Emotional reactions triggered by external situations.

(1) Clinician-Related Situational Responses

Patients’ perception of a medical institution’s professionalism can strengthen their trust and confidence in treatment. Positive clinician-patient interactions—including personalized care and encouragement to participate in treatment decisions—help patients build a psychology of feeling medically supported. However, when certain clinical plans (e.g., concurrent intravenous infusion and water restriction) conflict with patients’ basic physiological needs, psychological stress and negative emotions may arise.

*“Because I know this illness isn’t a big deal here at our Gulou Hospital.”* (P04)

*“I have confidence in my recovery—the nurses and doctors all care about us this much.”* (P06)

*“The IV infusion starts at 7 a.m. and goes on until 3 p.m. I just keep thinking: they won’t let me drink water, but they’re still giving me so much IV fluid.”* (P06)

(2) Family-Related Situational Responses

These refer to patients’ psychological reactions to family as an external emotional support system, including fears about poor prognosis arising from family responsibilities and emotional distress due to separation from relatives.

*“I think about my two kids—they’re in their early 20s. What if I can’t move anymore?”* (P03)

*“I miss my wife and kids.”* (P06)

(3) Responses Related to Reliance on Medical Technology

These encompass feelings of security derived from technical support, or anxiety due to its absence, reflecting patients’ psychological dependency on medical technology and the clinical environment as external safety conditions.

*“Now that I have the pacemaker implanted, I feel at ease—I can sleep whenever I want.” “What I’m afraid of right now is changes in my heart rate. If I don’t have a defibrillator, there’s nothing to provide protection for me. Even if I get one and might not even use it, I’ll still feel safer in my heart.”* (P08)

*“If they let me go home, I think I’ll still feel a bit scared to leave the hospital.”* (P08)

### 3.3 Theme 3: Cognitive dimension of symptom experience

This theme examines the cognitive aspects of postoperative symptom experience in valvular heart disease patients. It includes two facets: first, transient cognitive alterations (disorientation, hallucinations, misidentification, delusions) emerging in the early ICU phase, potentially anesthetic-induced, lasting 2–5 days and resolving by interview. Second, patients’ distinct symptom perceptions: viewing pain/fatigue as inevitable surgical outcomes, variable tolerance (e.g., pain vs. thirst), speculating age-symptom links (fatigue/anorexia), and anticipating post-discharge adjustment for appetite issues. Together, it highlights both transient neurocognitive impacts and patients’ active meaning-making of symptoms.

#### Subtheme 1: Experience of cognitive function changes.

Two patients exhibited postoperative changes in cognitive function, primarily characterized by cognitive disorientation, hallucinatory experiences, misidentification of others, and persecutory delusions. These alterations emerged in the early postoperative phase (during ICU stay). Anesthetic effects were identified as an initial trigger for cognitive changes in certain patients. The symptom duration was 2 days for Patient 01 and 5 days for Patient 14. Subsequently, both patients showed gradual symptom alleviation, and cognitive function had returned to normal by the time of interview.

*“There was a period when I felt really confused, especially after the anesthetic took effect. The symptoms were most obvious at night—it felt like I was trapped in a hallucinatory state.”* (P01)

*“I mistook other people for family members, and I couldn’t control myself... I heard someone saying that someone was coming to kill me, and my mind just wouldn’t calm down.”* (P14)

#### Subtheme 2: Cognition of symptoms.

Patients with valvular heart disease demonstrated distinctive perceptions regarding postoperative symptoms, reflected in the following aspects: a general belief that surgical trauma inevitably leads to symptoms such as pain and fatigue; varying tolerance levels for different symptoms (e.g., pain vs. thirst); attempts to explore potential correlations between age and symptoms such as fatigue and anorexia; and a tendency to anticipate long-term adjustment after discharge when facing appetite-related issues.

*“Later, I thought—this surgery cuts from the outside to the inside, so it must hurt. When I think about it, how could it not hurt?”* (P10)

*“From our perspective, this is unavoidable too. If we take a knife and cut ourselves, that wound hurts for days—let alone such a big surgery.”* (P03)

*“This is normal too. If you have such a big surgery, you’ll definitely feel tired.”* (P05)

*“I can bear a little pain, but thirst is really unbearable.”* (P03)

*“Older people tend to have this feeling of fatigue and anorexia, right?”* (P06)

*“I can’t eat—there’s nothing I can do. I just have to go home and adjust slowly.”* (P12)

### 3.4 Theme 4: Symptom experience and coping strategies in the behavioral dimension

This theme delineates patients’ specific behavioral responses to symptom-induced distress, revealing a continuum of strategies from passive endurance to active self-management. It comprises two major categories: passive behaviors elicited by symptoms and proactive coping strategies employed by patients.

#### Subtheme 1: Passive behavioral manifestations triggered by symptoms.

Severe symptoms, particularly pain and palpitations, frequently compromise patients’ physical function, resulting in the forced interruption or modification of activities. These behaviors are inherently passive and reactive. For instance, P04 stated, *“When I feel flustered, I just lie down for a bit”*; this action represents a compulsory physiological response rather than a volitional choice. Similarly, P08’s mobility was significantly constrained by symptoms, as evidenced by the report, *“only being able to walk a little less when my wound hurts.”* A more extreme form of passive adaptation was described by P04: *“When I was in the ICU... coughing irritated my wound and caused pain, so I could only press this area of the wound with my fingers.”* This instinctive self-protective measure underscores patient vulnerability and the restrictive impact of acute symptoms in the absence of immediate medical intervention.

#### Subtheme 2: Active coping strategies for symptoms.

In contrast to passive responses, patients also demonstrated substantial initiative in implementing active coping mechanisms. These strategies are broadly classified into three types: independent non-pharmacological coping, pharmacological and medical intervention coping, and support-seeking behaviors.

(1) Independent Non-Pharmacological Coping

Patients commonly employed a range of self-management strategies to alleviate discomfort. To manage thirst, P03 exhibited willpower-based endurance and quantitative control (*“If I prepare a small amount, I’ll drink less”*), whereas P14 adopted a functional alternative behavior (*“When I’m thirsty, I eat ice cubes”*). Coping strategies for insomnia varied considerably, encompassing ineffective cognitive efforts (P06: *“I started counting”*), sleep initiation contingent upon physical exhaustion (P07: *“When I’m exhausted and extremely sleepy, I’ll definitely fall asleep”*), and an attitude of acceptance framed as *“going with the flow”* (P13). Regarding pain management, some patients (e.g., P14) emphasized tolerance (*“I can bear the pain; I don’t want to take painkillers”*), while others engaged in positional adjustments (P03: *“I turn my body a little and use the pillow to slightly protect the wound”*) or pursued proactive methods such as ambulation (P11: *“Walking around and doing some exercise—recovery can help relieve chest tightness a bit”*).

(2) Pharmacological and Medical Intervention Coping

Patients’ approaches to medical intervention were characterized by a combination of adherence and autonomous judgment. They typically complied with medical advice when perceived as effective, resulting in significant symptom relief. For example, after analgesic administration, P02 reported, *“that night I slept well.”* Conversely, interventions deemed ineffective were proactively discontinued, as illustrated by P06: *“The sleeping pills didn’t work on the first day, so I stopped taking them the next day.”* Similarly, negative feedback from healthcare providers prompted behavioral adjustment; after being advised that excessive fluid intake exacerbated shortness of breath, P07 stated, *“Later I didn’t dare to drink water anymore, and I felt much better.”* Furthermore, patients sometimes initiated personalized modifications to prescribed regimens, demonstrating self-efficacy within a framework of general adherence. A representative example is P01’s approach to using Chuanbei Loquat Syrup: *“drink a little, not too much.”*

(3) Support-Seeking Coping from Others

Social support constituted a critical component of symptom management. Patients addressed physical symptoms, such as difficulty expectorating, by soliciting physical assistance from family members (P06: *“ask my family to pat my back”*) or by accessing professional care (P10: *“after I mentioned it to the nurse, the nurse helped me pat my back to clear phlegm”*). Beyond practical aid, emotional companionship itself served as an effective coping mechanism. For instance, the presence of family mitigated psychological distress for P14, who experienced postoperative cognitive impairment: *“my daughter came into the ICU to stay with me, and I felt better once she was there.”* This highlights the therapeutic role of intimate relationships in alleviating the illness experience.

## 4 Discussion

This qualitative study offers a nuanced exploration of symptom experiences and coping strategies among patients following cardiac valve surgery, illuminating the multisystemic, dynamic, and individually contingent nature of postoperative recovery. By centering patients’ lived experiences, this research not only corroborates existing knowledge of postoperative symptoms but also uncovers understudied psychological adaptations, cognitive shifts, and behavioral responses that shape rehabilitation trajectories. Below, we contextualize these findings within extant literature, highlight their clinical and theoretical implications, and address limitations and future research directions.

### 4.1 Diversity of Postoperative Physical Symptoms: Dynamics, Interactions, and Clinical Relevance

Participants’ physical symptom experiences underscore the profound multisystem impact of cardiac valve surgery, reflecting complex physiological processes—including surgical trauma, inflammatory cascades, and organ compensatory mechanisms. These symptoms were not isolated but interconnected, evolving dynamically across recovery phases and interacting to influence holistic well-being.

#### 4.1.1 Respiratory discomfort: From surgical trauma to adaptive recovery.

Consistent with prior research, respiratory symptoms—irritative cough, chest tightness, and dyspnea—predominated in the early postoperative period [12,13]. Cough is the most prevalent clinical manifestation, with previous studies reporting an incidence rate exceeding 80%. Postoperative cough typically reaches its peak incidence between postoperative days 3–5 [14], a finding corroborated by our current investigation.Participants described position-dependent coughing and phlegm retention, aligning with mechanisms such as endotracheal intubation-induced airway irritation, reduced functional residual capacity, and pleural effusion impairing gas exchange [13]. Dyspnea, primarily attributed to heart failure and pulmonary hypertension, is a typical preoperative symptom in patients with valvular heart disease [15], and it often persists or recurs postoperatively as a common manifestation of multiple pulmonary complications (including atelectasis, pleural effusion, and pneumonia). In the present study, pleural effusion was confirmed in patients who developed dyspnea. Notably, some attributed lingering cough to smoking withdrawal, emphasizing the need to differentiate surgical sequelae from pre-existing habits during symptom assessment.

#### 4.1.2 Discomfort in the pharynx and oral cavity: A multifactorial postoperative symptom complex.

Pharyngeal and oral discomfort is a common postoperative symptom cluster following cardiac valve surgery, comprising four subtypes: pharyngeal irritation, thirst, oral ulcers, and dysphagia with a choking sensation. Pharyngeal irritation manifests primarily as pharyngeal dryness, sore throat, hoarseness, and foreign body sensation, and typically resolves spontaneously within a few days [16]. Thirst, characterized by an intense need for fluids, exhibits a remarkably high prevalence after cardiac surgery and significantly impairs patients’ experience and quality of life [17]; indeed, several patients in this study described it as the most unbearable symptom. Oral ulcers, though less frequent, cause substantial swallowing pain due to mucosal barrier disruption, perioperative vitamin deficiencies (e.g., B-complex, vitamin C), and ICU-related mechanical friction. If untreated, these ulcers may delay recovery [18]. Dysphagia with a choking sensation, one of the most distressing subtypes, is often self-managed by patients through strategies such as slowing their eating pace and reducing portion sizes. A recent study reported that 14.8% of patients undergoing cardiac surgery experienced dysphagia [19], a finding consistent with our observation that this symptom was prevalent in our cohort.

Traditional clinical thinking often frames these symptoms as isolated events: sore throat is typically attributed to endotracheal intubation. However, a growing body of indirect evidence suggests that these four symptoms—sore throat, thirst, oral ulcers, and dysphagia with choking sensation—not only exhibit a high incidence in patients following cardiac valve surgery but also demonstrate significant overlap in onset time, potential triggers, and mutual pathophysiological influence [20–22]. For instance, prolonged endotracheal intubation is not only a direct cause of postoperative sore throat but also a key risk factor for inducing dysphagia. Additionally, thirst resulting from inadequate postoperative fluid replacement, coupled with xerostomia, can exacerbate pharyngeal irritation and may compromise the mucosal barrier function, thereby elevating the risk of oral ulcers. This phenomenon implies that conceptualizing these symptoms as an interconnected “symptom cluster” may offer greater clinical guidance than managing each symptom in isolation.

#### 4.1.3 Circulatory symptoms: Arrhythmias, palpitations, and the mind-body feedback loop.

Palpitation, defined as an unpleasant awareness of abnormal cardiac rhythm (e.g., tachycardia, bradycardia, irregularity, or forceful beats), is one of the most common chief complaints among postoperative patients and often indicates underlying arrhythmias. Postoperative arrhythmias—particularly atrial fibrillation (AF), the most frequent subtype—are closely associated with palpitation and represent a major clinical concern: they not only prolong hospital stays and increase medical costs but also correlate with severe adverse outcomes, including hemodynamic instability, exacerbated heart failure, elevated thromboembolic risk (especially stroke), and even sudden cardiac death [23,24]. This phenomenon driven by key pathophysiological mechanisms: myocardial ischemia (which disrupts atrial electrical remodeling), electrolyte imbalances (e.g., hypokalemia, which impairs cardiac conduction), and sympathetic overactivation (which increases atrial excitability) [25–27].

For participants like P02 (“My heart felt like it was leaping from my chest”), palpitations exacerbated fatigue and anxiety, creating a self-perpetuating cycle where physical symptoms fuel psychological distress and vice versa. This interplay necessitates holistic management: pharmacological control of arrhythmias paired with psychological support to mitigate anxiety-related tachycardia. As one participant noted, “Walking eases chest tightness,” graded activity tailored to cardiorespiratory fitness could serve dual roles—physical rehabilitation and psychological coping.

#### 4.1.4 Gastrointestinal and excretory issues: Nutritional recovery and hydration dilemmas.

Reduced appetite, a major barrier to postoperative recovery, can result in insufficient caloric intake, muscle atrophy, and immunosuppression. These issues not only prolong hospital stays but also increase mortality rates, with up to 25.7% of malnourished patients dying [28].

The causes of appetite loss are multifactorial, including inflammatory responses [29], cardiac insufficiency, mechanical ventilation effects, and psychological distress from anxiety or depression [30]. This study shows that most patients recognize the importance of postoperative nutritional support, collaborate actively with healthcare teams, and adapt individualized strategies to improve their nutritional status. Therefore, healthcare providers should conduct early nutritional risk assessments and implement multidisciplinary interventions tailored to each patient’s condition.

#### 4.1.5 Pain, fatigue, and sleep disturbances: A vicious cycle of interrelated symptoms.

Postoperative pain presents diverse manifestations (cramping, stabbing, burning) triggered by activity or position changes. The intensity and duration of pain vary from person to person but are universally prevalent. Studies have shown that many patients still experience moderate to severe pain for weeks or even months after surgery [31]. Frailty is prevalent among patients undergoing cardiac valve surgery; a study demonstrated that approximately 18% of patients presented with frailty at discharge [32]. Studies have reported that approximately 50% of patients undergoing cardiac surgery experience sleep disorders during hospitalization, and this problem may persist for weeks, months, or even longer [33].

These three symptoms are not mutually exclusive; instead, they form an intertwined, complex vicious cycle that severely hinders patients’ functional recovery, mental health, and overall well-being. Postoperative pain acts as a direct barrier to early mobilization and sleep [31]. Frailty, induced by physiological and psychological stress, impairs patients’ capacity to manage pain and facilitate recovery [32]. Furthermore, sleep deprivation or poor sleep quality not only amplifies pain perception but also delays tissue repair and may trigger or exacerbate negative emotions such as depression and anxiety [34].Our study has also identified the phenomenon of the intertwining of these symptoms.

### 4.2 Psychological adaptation: Resilience, distress, and social contextual influences

Existing literature predominantly emphasizes negative emotional outcomes (e.g., anxiety, depression) following cardiac surgery. In contrast, our study refines the conceptualization of psychological adaptation by elucidating its dual-level structure: an intrinsic psychological adaptation state that emerges spontaneously without direct external triggers, and emotional responses directly elicited by situational factors. This framework advances the understanding of postoperative psychological resilience and distress while elucidating the regulatory role of external contexts.

The intrinsic psychological adaptation state encompasses two opposing dimensions, reflecting patients’ inherent resilience or distress after surgery. This aligns with Leventhal’s Common-Sense Model, which posits that illness meaning-making buffers anxiety [35,36]. Specifically, positive intrinsic adaptation manifests as spontaneously generated positive emotions (e.g., hope, gratitude) and constructive cognitions (e.g., “I can recover with time”), reflecting intrinsic psychological resilience that operates independently of external support. This expands prior research focused on support-dependent positive emotions, highlighting the proactive role of patients’ internal resources in adaptation.

Conversely, negative intrinsic adaptation is characterized by spontaneous negative emotions (e.g., fear, existential distress), primarily driven by limited understanding of postoperative symptoms, concerns about surgical risks, or physiological changes (e.g., pain, fatigue). This challenges existing studies that attribute distress solely to objective clinical factors (e.g., symptom severity), revealing the intrinsic psychological mechanisms underlying postoperative distress.

External situational factors regulate psychological adaptation through three dimensions. Regarding clinician-related responses, patients’ perception of institutional professionalism and positive clinician-patient interactions enhance treatment trust, whereas conflicting clinical plans with basic physiological needs (e.g., pain management delays) may trigger stress. For family-related responses, family presence provides emotional support, reducing distress, while caregiving responsibilities or separation induce negative emotions (e.g., guilt, loneliness). As for technology dependence, medical devices (e.g., pacemakers) offer security but may provoke anxiety due to concerns about device failure or technical dependency.

This tripartite framework underscores the interplay between internal resilience and external stressors, emphasizing that psychological adaptation is neither static nor context-independent.

### 4.3 Coping strategies: The correlation with cognitive dimension of symptom experience and clinical implications

Valvular heart disease patients show distinct variability in postoperative coping strategies, which are shaped by symptom severity, personal beliefs, and social resources [37,38] and closely correlated with the cognitive dimension of symptom experience identified in our results. This supplements existing literature on cardiac postoperative coping mechanisms that inadequately link cognitive perceptions to coping behaviors [39]; notably, it highlights the guiding role of symptom cognitive interpretation in strategy selection.

Patients’ symptom cognitive recognition directly influences coping orientations. Those perceiving postoperative pain and fatigue as inevitable surgical trauma outcomes exhibit dual tendencies of passive endurance and active adaptation. Passive endurance against severe symptoms reflects vulnerability and temporary loss of functional autonomy [37], consistent with the view that postoperative passive coping is a defensive response to uncontrollable symptoms. In contrast, for symptoms with variable individual tolerance (e.g., mild pain), patients proactively explore adaptive methods (e.g., adjusting activity levels), reflecting attempts to balance symptom relief and medical norms.

Notably, early postoperative transient cognitive changes (e.g., disorientation) potentially affect coping effectiveness by impairing information processing and decision-making abilities. Patients with cognitive impairment rely more on passive coping (e.g., depending on nurses for daily care) and external support, transitioning to active coping as cognitive function recovers [40]. This dynamic process enriches understanding of the time-dependent characteristics of postoperative coping, emphasizing that coping strategies are not static but evolve with recovery.

The correlation between symptom cognitive dimension and coping strategies has important clinical implications, emphasizing personalized and integrated care. Clinically, comprehensive assessment of symptom severity (e.g., using the Visual Analog Scale for pain), coping preferences (e.g., via the Coping Inventory for Stressful Situations), symptom cognitive perception (e.g., using the Illness Perception Questionnaire), and cognitive function (e.g., via the Mini-Mental State Examination) is essential [41]. Targeted empowerment of self-management capabilities is required: for passive copers, proactive intervention (e.g., cognitive-behavioral therapy to reduce helplessness) is needed; for active copers, evidence-based non-pharmacological technique training (e.g., relaxation exercises for pain management) should be provided. Meanwhile, monitoring early postoperative transient cognitive changes (e.g., daily orientation assessments) and providing timely psychological support (e.g., counseling for anxiety) are necessary to avoid adverse outcomes [42]. Finally, integrating physical symptom intervention (e.g., medication for pain) and cognitive guidance (e.g., education on symptom meaning-making) helps establish scientific symptom understanding and forms a synergistic effect of physical and psychological care.

## 5 Conclusion

This study illuminates the multifaceted symptom experiences of post – cardiac valve surgery patients, emphasizing the interplay of physical, psychological, cognitive, and behavioral factors. By centering patient voices, we highlight the need for holistic, individualized care—addressing not just symptoms, but their origins and social contexts. Ultimately, this research provides a foundation for developing targeted interventions to improve outcomes and quality of life.

## 6 Limitations

This study has three primary limitations: (1) a homogenous small sample from a single Chinese tertiary hospital may limit the statistical power and generalizability of our findings;(2) exclusive reliance on retrospective self-reporting may introduce selection bias and recall errors;(3) the considerable variation observed in our data makes it challenging to draw definitive conclusions about our study.(4) The reliance on retrospective interviews may introduce recall bias, potentially limiting the accuracy of capturing participants’ real-time experiences and symptom progression during hospitalization.To address these limitations, future research should employ mixed-methods approaches with diverse populations, including international cohorts, to validate the current findings. Incorporating wearable biosensors for real-time symptom monitoring could enhance the objectivity of data collection, complementing subjective self-reports. Additionally, longitudinal studies tracking patients at multiple intervals over the first postoperative year are essential. These studies should focus on exploring symptom trajectories in relation to key biopsychosocial recovery milestones, offering a more comprehensive understanding of the postoperative recovery process.

## Supporting information

S1 File The complete list of interview questions. (DOCX)

S2 Fig Thematic evolution pathway diagram. (DOCX)
