# Supplementary material for: Symptom experiences of patients after cardiac valve surgery: A qualitative study
Source: PLoS One. 2026 Mar 10;21(3):e0342597. doi: 10.1371/journal.pone.0342597 (PMC12974852; doi:10.1371/journal.pone.0342597)
Supplement: S1 File — (DOCX) [file pone.0342597.s001.docx]

The complete list of interview questions :

①Since you had the cardiac valve surgery, could you describe in detail all the physical discomforts or unusual feelings you have noticed? For example, do you have chest tightness, shortness of breath, fatigue, or other sensations that you didn't have before the surgery?

②Over time, have you observed any changes in the symptoms? For example, have they become more severe, less severe, or have new symptoms emerged while some old ones disappeared?

③How have these symptoms affected your daily life activities, such as dressing, bathing, or mobility?

④Have the symptoms had any influence on your emotional state, such as making you feel anxious, depressed, frustrated, or worried?

⑤When the post - surgery symptoms (such as fatigue, shortness of breath, or chest tightness) occur, have you tried adjusting your daily life habits to relieve them? For example, changing your sleep schedule (like taking naps), adjusting your diet (such as reducing salt or fat intake), or modifying your daily activity rhythm (like slowing down the pace of doing things)? If yes, could you describe the specific adjustments you made and how you decided to try these methods?

⑥The doctor may have prescribed medications to manage post - surgery symptoms (such as painkillers for chest pain or drugs to relieve shortness of breath). How do you use these medications to cope with symptoms? For example, do you take them strictly according to the doctor's instructions when symptoms appear, or do you adjust the dosage slightly based on your own feelings? Have you noticed any effects after taking the medications, and are there any side effects that make you uncomfortable?

⑦The symptoms you’ve experienced after surgery (like chest tightness, fatigue, or palpitations) have impacted your daily life. What expectations do you have for the improvement or disappearance of these symptoms?
