# Supplementary material for: Symptom experiences of patients after cardiac valve surgery: A qualitative study
Source: PLoS One. 2026 Mar 10;21(3):e0342597. doi: 10.1371/journal.pone.0342597 (PMC12974852; doi:10.1371/journal.pone.0342597)
Supplement: S2 Fig — (DOCX) [file pone.0342597.s002.docx]

| thematic evolution pathway diagram | | |
| --- | --- | --- |
| P01"On the one hand, coughing is the simplest and most prominent symptom. For example, if you sit in an incorrect posture or keep shifting positions, or talk for a long time, you will start coughing immediately. " | Cough | Respiratory discomfort |
| P04 "I used to smoke. If I go days without one, I might also get withdrawal symptoms like this cough""It's already better now, the symptoms have subsided" |  |  |
| P05"The cough has lasted for four to five days, and it is slightly better now" |  |  |
| P06 "The cough symptoms should be getting lighter and lighter." |  |  |
| P08"Two days after being transferred out of the care unit, I felt the urge to cough. It was just a dry cough with no sputum" |  |  |
| P10"Sputum couldn’t be coughed up—there was sputum, but it was difficult to expectorate, and the sputum that came out was like mucus""After I cough, both sides of my back hurt" |  |  |
| P12 "Coughing occurs during nebulization" |  |  |
| P13 "A few days later after surgery, there was phlegm and coughing." |  |  |
| P07 I've been feeling a bit short of breath for the past couple of days, and it just started. It began to feel this way around a week after the surgery. | Shortness of breath |  |
| P05 When urination is reduced, I feel chest tightness, and then feel uncomfortable all over my body. Today, the doctor also did an ultrasound, which showed a small amount of pleural effusion. The doctor said that this situation can also cause chest tightness. | Chest tightness |  |
| P11 Often feel chest tightness |  |  |
